# Supplementary material for: The Aquilegia genome provides insight into adaptive radiation and reveals an extraordinarily polymorphic chromosome with a unique history
Source: eLife. 2018 Oct 16;7:e36426. doi: 10.7554/eLife.36426 (PMC6255393; doi:10.7554/eLife.36426)
Supplement: Supplementary file 1. [file elife-36426-supp1.pdf]

Supplementary File 1. Genomic libraries included in the *A. coerulea* genome assembly and their respective assembled sequence coverage levels in *A. coerulea* v3.1 release.

Library	Sequencing platform	Average read/ insert size	Read number	Assembled equence overage (x)
FTOY	Sanger	2,632 ± 200	1,609,053	2.56
GHCH	Sanger	2,638 ± 201	489,984	0.80
FTOX	Sanger	6,458 ± 677	1,804,031	2.87
GHHF	Sanger	6,460 ± 679	519,264	0.83
FTOW	Sanger	33,419 ± 3,521	219,070	0.31
GGNB	Sanger	33,431 ± 3,496	36,768	0.05
COL	Sanger	123,731 ± 38,119	95,040	0.17
Total			4,773,210	7.59
