# Supplementary material for: The Aquilegia genome provides insight into adaptive radiation and reveals an extraordinarily polymorphic chromosome with a unique history
Source: eLife. 2018 Oct 16;7:e36426. doi: 10.7554/eLife.36426 (PMC6255393; doi:10.7554/eLife.36426)
Supplement: Supplementary file 2. [file elife-36426-supp2.pdf]

Supplementary File 2. Summary statistics of the output of the whole genome shotgun assembly prior to screening, removal of organelles and contaminating scaffolds and chromosome-scale pseudomolecule construction. The table shows total contigs and total assembled basepairs for each set of scaffolds greater than the size listed in the left hand column.

Minimum scaffold length	Number of scaffolds	Number of contigs	Scaffold size	Basepairs	% Non-gap basepairs
5 Mb	14	1,438	94,320,677	92,114,319	97.66
2.5 Mb	38	2,890	177,446,204	172,758,974	97.36
1 Mb	87	4,445	257,833,655	250,413,462	97.12
500 Kb	119	5,059	282,450,696	273,716,964	96.91
250 Kb	140	5,324	290,151,377	280,639,834	96.72
100 Kb	168	5,631	294,581,469	283,539,175	96.25
50 Kb	194	5,808	296,290,157	284,968,083	96.18
25 Kb	255	6,094	298,499,112	286,606,915	96.02
10 Kb	606	7,055	303,523,673	291,234,712	95.95
5 Kb	1,202	8,310	307,762,011	295,088,014	95.88
2.5 Kb	2,152	9,914	311,235,474	298,320,877	95.85
1 Kb	2,226	10,013	311,372,308	298,443,577	95.85
0 bp	2,529	10,316	311,527,937	298,599,206	95.85
