# Supplementary material for: The Aquilegia genome provides insight into adaptive radiation and reveals an extraordinarily polymorphic chromosome with a unique history
Source: eLife. 2018 Oct 16;7:e36426. doi: 10.7554/eLife.36426 (PMC6255393; doi:10.7554/eLife.36426)
Supplement: Supplementary file 3. [file elife-36426-supp3.pdf]

Supplementary File 3. Final summary assembly statistics for chromosome-scale assembly.

Scaffold total	1,034
Contig total	7,930
Scaffold sequence total	306.5 Mb
Chromosome sequence	282.6 Mb
Contig sequence total	291.7 Mb (4.8% gap)
Scaffold N/L50	4/43.6 Mb
Contig N/L50	797/110.9 Kb
