# Supplementary material for: The Aquilegia genome provides insight into adaptive radiation and reveals an extraordinarily polymorphic chromosome with a unique history
Source: eLife. 2018 Oct 16;7:e36426. doi: 10.7554/eLife.36426 (PMC6255393; doi:10.7554/eLife.36426)
Supplement: Supplementary file 4. [file elife-36426-supp4.pdf]

Supplementary File 4. Placement of the individual BAC clones and their contribution to the overall error rate.

Fosmid clone ID	Length	Chromosome	Start	Stop	Discrepant bases
9413	119658	Chr_02	42736342	42855946	0
9446	173129	Chr_02	20577579	20750820	1
9436	124874	Chr_05	34205168	34328207	2
9431	45731	Chr_06	10559008	10602180	1
9449	183288	Chr_01	2756617	2939634	18
9412	166127	Chr_07	17866735	18030762	33
9419	130227	Chr_03	19399302	19529716	28
9437	119406	Chr_01	36503268	36621202	27
9429	131322	Chr_05	15731088	15862409	35
9450	121062	Chr_05	42742789	42865380	36
9428	137275	Chr_01	18470512	18607862	41
9404	107650	Chr_03	32268312	32374757	35
9427	160464	Chr_07	543990	704396	53
9442	130064	Chr_03	30119597	30129597	48
9416	167046	Chr_05	23092053	23258725	69
9409	140373	Chr_07	31933377	32073020	72
9435	117612	Chr_04	44491410	44609808	63
9403	153476	Chr_02	38659429	38811278	98
9441	87456	Chr_04	6547153	6628689	78
9408	165608	Chr_07	30512052	30676049	236
9444	171059	Chr_04	25447280	25613428	363
9447	65772	Chr_06	1652383	1717537	151
9433	145126	Chr_04	38529772	38643947	343
Total	3,063,805				
