# Supplementary material for: The Aquilegia genome provides insight into adaptive radiation and reveals an extraordinarily polymorphic chromosome with a unique history
Source: eLife. 2018 Oct 16;7:e36426. doi: 10.7554/eLife.36426 (PMC6255393; doi:10.7554/eLife.36426)
Supplement: Supplementary file 6. [file elife-36426-supp6.pdf]

Supplementary File 6. Ratio of polymorphism (poly) or divergence (div) on each chromosome versus genome-wide for each species. Polymorphism is the number of pairwise differences within a species, divergence is the pairwise differences between the species and Semiaquilegia. Both are calculated at fourfold degenerate sites.

Species	Ratio type	Chr_01	Chr_02	Chr_03	Chr_04	Chr_05	Chr_06	Chr_07
A. pubescens	poly	0.310	1.154	1.167	1.774	1.058	1.109	1.112
	div	0.971	0.973	1.004	1.204	0.972	1.014	1.008
A. barnebyi	poly	0.915	0.868	0.979	2.053	0.968	0.958	0.979
	div	0.974	0.973	1.004	1.206	0.972	1.016	1.003
A. aurea	poly	0.956	0.790	0.807	1.601	1.121	1.834	0.450
	div	0.977	0.969	1.006	1.190	0.980	1.010	1.002
A. vulgaris	poly	0.920	1.239	0.693	1.926	0.806	1.025	1.091
	div	0.977	0.971	1.005	1.189	0.982	1.008	1.001
A. sibirica	poly	0.681	0.436	2.489	1.055	1.034	0.317	0.838
	div	0.979	0.970	1.003	1.205	0.979	1.004	1.003
A. formosa	poly	0.958	0.996	1.020	1.641	0.927	0.879	1.003
	div	0.976	0.973	1.004	1.205	0.970	1.012	1.006
A. japonica	poly	0.931	1.031	0.884	1.790	0.984	1.080	0.868
	div	0.977	0.962	1.005	1.204	0.984	1.008	1.002
A. oxysepala	poly	1.627	0.661	0.542	6.182	0.495	0.350	0.424
	div	0.974	0.969	1.001	1.192	0.987	1.007	1.006
A. longissima	poly	1.630	0.649	0.691	2.780	0.872	0.486	0.869
	div	0.974	0.971	1.001	1.205	0.977	1.011	1.005
A. chrysantha	poly	0.936	1.051	0.979	1.776	0.928	0.913	0.946
	div	0.975	0.972	1.002	1.211	0.974	1.015	1.002
mean	poly	0.986	0.887	1.025	2.258	0.919	0.895	0.858
	div	0.975	0.970	1.004	1.201	0.978	1.011	1.004
