# Supplementary material for: The Aquilegia genome provides insight into adaptive radiation and reveals an extraordinarily polymorphic chromosome with a unique history
Source: eLife. 2018 Oct 16;7:e36426. doi: 10.7554/eLife.36426 (PMC6255393; doi:10.7554/eLife.36426)
Supplement: Supplementary file 7. [file elife-36426-supp7.pdf]

Supplementary File 7. Robustness of nucleotide diversity patterns to copy number variant detection methods.

		Percent nucleotide diversity							
		Chromosome							
Method	Species	1	2	3	4	5	6	7	Genome
0.15log cov	*A. pubescens*	0.027	0.097	0.092	0.151	0.098	0.092	0.096	0.084
	A. barnebyi	0.053	0.055	0.046	0.086	0.050	0.046	0.047	0.051
	A. aurea	0.009	0.005	0.005	0.001	0.012	0.017	0.001	0.007
	A. vulgaris	0.103	0.120	0.071	0.156	0.087	0.103	0.113	0.101
	A. sibirica	0.018	0.005	0.084	0.033	0.025	0.005	0.017	0.027
	A. formosa	0.123	0.112	0.116	0.165	0.111	0.122	0.112	0.118
	A. japonica	0.144	0.152	0.143	0.247	0.149	0.151	0.144	0.151
	A. oxysepala	0.042	0.005	0.002	0.116	0.002	0.001	0.001	0.015
	A. longissima	0.029	0.007	0.008	0.037	0.010	0.003	0.013	0.014
	A. chrysantha	0.097	0.106	0.088	0.161	0.097	0.087	0.095	0.098
CNV detection	*A. pubescens*	0.094	0.332	0.316	0.446	0.289	0.293	0.290	0.264
	A. barnebyi	0.196	0.195	0.182	0.351	0.201	0.197	0.192	0.198
	A. aurea	0.030	0.025	0.023	0.017	0.025	0.055	0.013	0.027
	A. vulgaris	0.225	0.295	0.132	0.343	0.190	0.241	0.245	0.223
	A. sibirica	0.055	0.020	0.169	0.034	0.091	0.028	0.061	0.072
	A. formosa	0.305	0.316	0.317	0.436	0.301	0.307	0.316	0.314
	A. japonica	0.307	0.305	0.275	0.551	0.309	0.345	0.296	0.312
	A. oxysepala	0.099	0.033	0.027	0.251	0.020	0.015	0.018	0.046
	A. longissima	0.079	0.032	0.026	0.107	0.033	0.022	0.040	0.043
	A. chrysantha	0.306	0.318	0.282	0.421	0.305	0.301	0.305	0.306
Tandem duplicates	*A. pubescens*	0.082	0.340	0.324	0.473	0.322	0.288	0.305	0.282
	A. barnebyi	0.191	0.197	0.191	0.364	0.200	0.176	0.190	0.200
	A. aurea	0.029	0.027	0.024	0.035	0.043	0.046	0.016	0.031
	A. vulgaris	0.214	0.293	0.164	0.403	0.198	0.217	0.238	0.228
	A. sibirica	0.054	0.033	0.184	0.103	0.073	0.030	0.063	0.077
	A. formosa	0.321	0.325	0.320	0.483	0.318	0.309	0.321	0.328
	A. japonica	0.296	0.314	0.287	0.571	0.299	0.317	0.296	0.314
	A. oxysepala	0.102	0.037	0.027	0.319	0.023	0.018	0.022	0.055
	A. longissima	0.079	0.030	0.037	0.137	0.039	0.018	0.041	0.047
	A. chrysantha	0.304	0.329	0.291	0.496	0.302	0.298	0.312	0.315
Allele ratio in reads	*A. pubescens*	0.045	0.201	0.254	0.471	0.321	0.289	0.305	0.241
	A. barnebyi	0.139	0.135	0.155	0.370	0.198	0.179	0.190	0.176
	A. aurea	0.012	0.007	0.012	0.039	0.044	0.047	0.018	0.023
	A. vulgaris	0.162	0.224	0.127	0.408	0.204	0.219	0.241	0.204
	A. sibirica	0.031	0.010	0.153	0.108	0.074	0.030	0.066	0.065
	A. formosa	0.243	0.230	0.273	0.476	0.318	0.308	0.320	0.290
	A. japonica	0.225	0.234	0.247	0.579	0.298	0.320	0.297	0.283
	A. oxysepala	0.066	0.009	0.012	0.329	0.025	0.019	0.024	0.043
	A. longissima	0.057	0.016	0.027	0.139	0.040	0.019	0.041	0.040
	A. chrysantha	0.226	0.232	0.249	0.495	0.303	0.296	0.310	0.279
