# Supplementary material for: The Aquilegia genome provides insight into adaptive radiation and reveals an extraordinarily polymorphic chromosome with a unique history
Source: eLife. 2018 Oct 16;7:e36426. doi: 10.7554/eLife.36426 (PMC6255393; doi:10.7554/eLife.36426)
Supplement: Supplementary file 8. [file elife-36426-supp8.pdf]

Supplementary File 8. Repeat family prevalence and permutation results in the *A. coerulea* v3.1 genome release.

Family	Observed copy number on Chr_04	Number of permutations with fewer	Number of permutations with more	Number of permutations with same
DNA CMC-EnSpm	443	1000	0	0
DNA TcMar-Pogo	637	1000	0	0
DNA hAT-Tip100	49	1000	0	0
LINE CRE-II	1109	1000	0	0
LINE L1	2038	1000	0	0
LINE Tad1	90	1000	0	0
LTR	377	1000	0	0
LTR Copia	4341	1000	0	0
LTR Gypsy	4097	1000	0	0
SINE tRNA	1173	1000	0	0
Unknown	28188	1000	0	0
LINE L2	26	994	2	4
LINE R1	52	998	2	0
DNA hAT-Ac	1376	991	9	0
DNA TcMar-Stowaway	29	951	26	23
DNA MuLE-MuDR	301	961	35	4
LTR Caulimovirus	148	945	46	9
Simple repeat	326	935	57	8
DNA Maverick	27	910	64	26
LTR Ngaro	13	864	68	68
Satellite	41	828	130	42
rRNA	42	811	145	44
DNA hAT	41	719	229	52
LTR ERVK	18	665	247	88
LINE RTE-BovB	14	579	305	116
snRNA	12	557	311	132
DNA MULE-MuDR	299	659	323	18
RC Helitron	135	340	624	36
DNA Crypton	11	250	641	109
SINE?	80	259	700	41
DNA PIF-Harbinger	270	135	848	17
DNA TcMar	51	102	863	35
LTR ERV1	63	48	942	10
DNA	16	2	992	6
DNA hAT-Tag1	381	6	993	1
DNA Sola	1	0	996	4
LINE L1-Tx1	4	0	999	1
DNA Dada	0	0	1000	0
