# Supplementary material for: The Aquilegia genome provides insight into adaptive radiation and reveals an extraordinarily polymorphic chromosome with a unique history
Source: eLife. 2018 Oct 16;7:e36426. doi: 10.7554/eLife.36426 (PMC6255393; doi:10.7554/eLife.36426)
Supplement: Supplementary file 9. [file elife-36426-supp9.pdf]

Supplementary File 9. K-mer based estimates of genome size and repetitive sequence proportion

Geographic region	Species	Genome size (bp)	Proportion repetitive
North America	*A. pubescens*	327739099	0.473
	A. formosa	320736307	0.442
	A. barnebyi	314243005	0.449
	A. longissima	315526747	0.452
	A. chrysantha	327663698	0.443
Europe	*A. aurea*	301759711	0.443
	A. vulgaris	298726981	0.419
Asia	*A. sibirica*	289831630	0.417
	A. flabellata	284394029	0.402
	A. oxysepala	316478823	0.452
