# Supplementary material for: The Aquilegia genome provides insight into adaptive radiation and reveals an extraordinarily polymorphic chromosome with a unique history
Source: eLife. 2018 Oct 16;7:e36426. doi: 10.7554/eLife.36426 (PMC6255393; doi:10.7554/eLife.36426)
Supplement: Supplementary file 10. [file elife-36426-supp10.pdf]

Supplementary File 10. Mean and median coverage by species.

Species	Mean coverage	Median coverage
A. pubescens	58	46
A. barnebyi	105	88
A. aurea	116	97
A. vulgaris	122	93
A. sibirica	112	91
A. formosa	122	100
A. japonica	124	100
A. oxysepala	116	87
A. longissima	116	94
A. chrysantha	116	94
Semiaquilegia adoxoides	74	31
