# Supplementary material for: The Aquilegia genome provides insight into adaptive radiation and reveals an extraordinarily polymorphic chromosome with a unique history
Source: eLife. 2018 Oct 16;7:e36426. doi: 10.7554/eLife.36426 (PMC6255393; doi:10.7554/eLife.36426)
Supplement: Supplementary file 11. [file elife-36426-supp11.pdf]

Supplementary File 11. Proportion of sites removed by each filter (sites can have more than one filter) by chromosome - for initial filtration without Semi.

Chr	HCov*	INDEL	JGIREP	MINCOV	MissCall	RepMask	MULTI	Unfiltered
Chr_01	0.096	0.216	0.373	0.447	0.309	0.029	0.002	0.264
Chr_02	0.101	0.212	0.394	0.476	0.311	0.028	0.002	0.227
Chr_03	0.099	0.214	0.375	0.462	0.291	0.029	0.002	0.245
Chr_04	0.076	0.230	0.520	0.702	0.461	0.028	0.003	0.071
Chr_05	0.099	0.215	0.379	0.464	0.301	0.029	0.002	0.250
Chr_06	0.103	0.213	0.377	0.478	0.330	0.030	0.002	0.240
Chr_07	0.110	0.212	0.389	0.486	0.328	0.029	0.002	0.220

*Hcov=coverage less than 0.5x log median coverage or greater than -0.5x log median coverage, INDEL= indel and +/-10bp, JGIREP=repetitive sequence, MINCOV= coverage <15, MissCall=call missing in any species, RepMask=removed by RepeatMasker, MULTI=multiallelic, Unfiltered=site retained in data
