# Supplementary material for: The Aquilegia genome provides insight into adaptive radiation and reveals an extraordinarily polymorphic chromosome with a unique history
Source: eLife. 2018 Oct 16;7:e36426. doi: 10.7554/eLife.36426 (PMC6255393; doi:10.7554/eLife.36426)
Supplement: Supplementary file 12. [file elife-36426-supp12.pdf]

Supplementary File 12. Proportion of sites removed by each filter (sites can have more than one filter) by chromosome - for final filtration with *Semiaquilegia*.

Chr	HCov*	INDEL	JGIREP	MINCOV	MissCall	RepMask	MULTI	Unfiltered
Chr_01	0.089	0.272	0.373	0.541	0.391	0.029	0.003	0.205
Chr_02	0.091	0.265	0.394	0.575	0.398	0.028	0.003	0.176
Chr_03	0.090	0.268	0.375	0.559	0.379	0.029	0.003	0.189
Chr_04	0.054	0.270	0.520	0.800	0.572	0.028	0.004	0.049
Chr_05	0.093	0.271	0.379	0.546	0.378	0.029	0.004	0.195
Chr_06	0.094	0.269	0.377	0.561	0.407	0.030	0.003	0.186
Chr_07	0.098	0.265	0.389	0.577	0.413	0.029	0.003	0.170

*Hcov=coverage less than 0.5x log median coverage or greater than -0.5x log median coverage, INDEL= indel and +/-10bp, JGIREP=repetitive sequence, MINCOV= coverage <15, MissCall=call missing in any species, RepMask=removed by RepeatMasker, MULTI=multiallelic, Unfiltered=site retained in data
