# Supplementary material for: The Aquilegia genome provides insight into adaptive radiation and reveals an extraordinarily polymorphic chromosome with a unique history
Source: eLife. 2018 Oct 16;7:e36426. doi: 10.7554/eLife.36426 (PMC6255393; doi:10.7554/eLife.36426)
Supplement: Supplementary file 13. [file elife-36426-supp13.pdf]

Supplementary File 13. Number of derived variants by species.

		Number of variants		
Geographic region	Species	Private	Shared	Total
Asia	*A. japonica*	129211	205724	334935
	A. oxysepala	134400	147923	282323
	A. sibirica	99369	176177	275546
Europe	*A. aurea*	107824	176951	284775
	A. vulgaris	139849	189526	329375
North America	*A. barnebyi*	75641	245644	321285
	A. chrysantha	87258	261239	348497
	A. formosa	87823	266604	354427
	A. longissima	66344	232710	299054
	A. pubescens	72661	265713	338374
