# Supplementary material for: The Aquilegia genome provides insight into adaptive radiation and reveals an extraordinarily polymorphic chromosome with a unique history
Source: eLife. 2018 Oct 16;7:e36426. doi: 10.7554/eLife.36426 (PMC6255393; doi:10.7554/eLife.36426)
Supplement: Supplementary file 14. [file elife-36426-supp14.pdf]

Supplementary File 14. Transition matrix for the Five-State Markov process. m_1 denotes the probability of an *A. oxysepala* lineage being a migrant, while m_2 denotes the probability of a *A. japonica* lineage being a migrant at a given generation.

	(1,1,1)	(1,2,0)	(1,0,2)	(1,0,1)	(1,1,1)*
(1,1,1)	1-(m_1+m_2)	m_1	m_2	0	0
(1,2,0)	m_2	1-(2*m_2+1/N)	0	1/N	m_2
(1,0,2)	m_1	0	1-(2*m_1+1/N)	1/N	m_1
(1,0,1)	0	0	0	1	0
(1,1,1)*	0	m_1	m_2	0	1-(m_1+m_2)
