# Supplementary material for: The Aquilegia genome provides insight into adaptive radiation and reveals an extraordinarily polymorphic chromosome with a unique history
Source: eLife. 2018 Oct 16;7:e36426. doi: 10.7554/eLife.36426 (PMC6255393; doi:10.7554/eLife.36426)
Supplement: Supplementary file 15. [file elife-36426-supp15.pdf]

Supplementary File 15. Transition matrix for the Eight-State Markov process. X=$(1\text{-}1/N)^T$ and Y=$(1\text{-}3/N)^T$

	(1,1,1)	(1,2,0)	(1,0,2)	(1,0,1)	(1,1,1)†	(0,1,1)	(1,1,0)	(1,1,1)*
(1,1,1)	0	0	0	0	X	1-X	0	0
(1,2,0)	0	0	0	(1-Y)/3	Y	(1-Y)/3	(1-Y)/3	0
(1,0,2)	0	0	0	1-X	X	0	0	0
(1,0,1)	0	0	0	1	0	0	0	0
(1,1,1)†	0	0	0	1/N	1-3/N	1/N	1/N	0
(0,1,1)	0	0	0	0	0	1	0	0
(1,1,0)	0	0	0	0	0	0	1	0
(1,1,1)*	0	0	0	0	X	0	1-X	0
